# Cancer Immunotherapy Employing an Innovative Strategy to Enhance CD4+ T Cell Help in the Tumor Microenvironment

**DOI:** 10.1371/journal.pone.0115711

**Published:** 2014-12-22

**Authors:** Liwen Song, Ming-Chieh Yang, Jayne Knoff, T.-C. Wu, Chien-Fu Hung

**Affiliations:** 1 Department of Obstetrics and Gynecology, Shanghai Tenth People's Hospital of Tongji University Shanghai, China; 2 Pharmacy School of Fudan University, Shanghai, China; 3 Department of Pharmacology and Toxicology, Shanghai Institute of Planned Parenthood Research, Shanghai, China; 4 Department of Pathology, Johns Hopkins Medical Institutions, Baltimore, MD, United States of America; 5 Department of Obstetrics and Gynecology, Johns Hopkins Medical Institutions, Baltimore, MD, United States of America; 6 Department of Molecular Microbiology and Immunology, Johns Hopkins Medical Institutions, Baltimore, MD, United States of America; 7 Department of Oncology, Johns Hopkins Medical Institutions, Baltimore, MD, United States of America; 8 General Surgery and Surgical Intensive Care, Kaohsiung Veterans General Hospital, Taiwan; Leiden University Medical Center, Netherlands

## Abstract

Chemotherapy and/or radiation therapy are widely used as cancer treatments, but the antitumor effects they produce can be enhanced when combined with immunotherapies. Chemotherapy kills tumor cells, but it also releases tumor antigen and allows the cross-presentation of the tumor antigen to trigger antigen-specific cell-mediated immune responses. Promoting CD4+ T helper cell immune responses can be used to enhance the cross-presentation of the tumor antigen following chemotherapy. The pan HLA-DR binding epitope (PADRE peptide) is capable of generating antigen-specific CD4+ T cells that bind various MHC class II molecules with high affinity and has been widely used in conjunction with vaccines to improve their potency by enhancing CD4+ T cell responses. Here, we investigated whether intratumoral injection of PADRE and the adjuvant CpG into HPV16 E7-expressing TC-1 tumors following cisplatin chemotherapy could lead to potent antitumor effects and antigen-specific cell-mediated immune responses. We observed that treatment with all three agents produced the most potent antitumor effects compared to pairwise combinations. Moreover, treatment with cisplatin, CpG and PADRE was able to control tumors at a distant site, indicating that our approach is able to induce cross-presentation of the tumor antigen. Treatment with cisplatin, CpG and PADRE also enhanced the generation of PADRE-specific CD4+ T cells and E7-specific CD8+ T cells and decreased the number of MDSCs in tumor loci. The treatment regimen presented here represents a universal approach to cancer control.

## Introduction

Chemotherapy and/or radiation therapy are widely used as cancer treatments. Both chemotherapy and radiation therapy have been shown to transform the tumor microenvironment into a suitable setting for subsequent immunotherapeutic vaccination [1], [2]. We have previously used cisplatin chemotherapy to prime the tumor microenvironment for vaccination with a recombinant protein, and found that this treatment regimen induced potent antitumor effects and antigen-specific cell-mediated immune responses [1]. Not only does cisplatin kill tumor cells but also it releases tumor antigen and allows the cross-presentation of the tumor antigen to trigger antigen-specific cell-mediated immune responses. However, the antitumor effects produced by chemotherapy can be enhanced when combined with immunotherapies.

A strategy to enhance the cross-presentation of the tumor antigen following chemotherapy is to promote CD4+ T helper cell immune responses. An agent capable of generating antigen-specific CD4+ T cells that bind various MHC class II molecules with high affinity is the pan HLA-DR binding epitope (PADRE peptide) [3]. The PADRE peptide has been widely used in conjunction with vaccines to improve their potency by enhancing CD4+ T cell responses [4]–[7]. Therefore, intratumoral administration of PADRE potentially can create PADRE-specific CD4+ T helper cells to further improve cross-presentation to generate tumor antigen-specific CD8+ T cells. The employment of an immunostimulatory adjuvant with PADRE peptide may further enhance tumor antigen-specific CD8+ T cells.

The toll-like receptor 9 (TLR9) agonist CpG is a commonly used adjuvant that has been shown to stimulate CD8+ T cell cross-priming by promoting type I interferon production [8], [9]. CpG has also been shown to have antitumor effects when directly injected into the tumor [10]–[12]. Furthermore, CpG has been shown to block the immunosuppressive activity of MDSCs in tumor-bearing mice [13]. These studies suggest that the immunostimulatory function of CpG can be used to enhance the cross-presentation of tumor antigen to generate tumor antigen-specific CD8+ T cell-mediated immune responses.

In the current study, we hypothesized that cisplatin treatment followed by CpG adjuvant and PADRE peptide administration would enhance the cross-presentation of tumor antigen, leading to potent antitumor effects. To test this, we used mice bearing HPV16 E7-expressing TC-1 tumors and treated them with various combinations of cisplatin followed by intratumoral injection with CpG and PADRE peptide. We found that treatment with all three agents produced the most potent antitumor effects. Moreover, treatment with cisplatin, CpG and PADRE was able to control tumors at a distant site, indicating that our approach was able to induce cross-presentation of the tumor antigen. We found that treatment with cisplatin, CpG and PADRE enhanced the generation of PADRE-specific CD4+ T cells as well as E7-specific CD8+ T cells. Treatment with cisplatin, CpG and PADRE also decreased the number of MDSCs in tumor loci, a process found to be mediated by the Fas-FasL apoptosis pathway. The treatment regimen presented here is a novel application of a combination of immunotherapies that induces potent antitumor immune responses without requiring knowledge of immunodominant tumor antigens, making the approach potentially widely applicable.

## Materials and Methods

### Ethics Statement

All animal procedures used in this study were performed according to protocols approved for this specific study and in accordance with recommendations for the proper use and care of laboratory animals by Johns Hopkins University Animal Care and Use Committee. All cell lines were established and maintained with approved protocols. Mice were sacrificed for the purpose of this study using CO_2_ in accordance with the animal protocol. Regarding human endpoint standards, mice showing severe distress or bearing tumors that exceeded 20mm in diameter were euthanized with CO_2_ in accordance with the animal protocol.

### Experimental animals

Six- to eight-week-old female C57BL/6 mice were obtained from the National Cancer Institute-Frederick Animal Production Area (Frederick, MD). Mice were housed in the oncology animal facility of the Johns Hopkins Hospital (Baltimore, MD).

### Cells

The TC-1 tumor model was produced in our laboratory by transformation of primary lung epithelial cells from C57BL/6 mice with active Ras together with HPV16 E6 and E7 oncogenes and the production and maintenance under approved protocols of this cell line has been described previously [14]. Cells were cultured in RPMI-1640 medium containing 10% FBS, 2 mM L-glutamine, 10% sodium pyruvate, 10% non-essential amino acid, and 100 pg/ml streptomycin in a humidified atmosphere of 5% CO_2_/95% air at 37°C. E7-specific CD8+ T cells were generated from splenocytes of E7 vaccinated mice and were stimulated with irradiated TC-1 cells and 10 IU interleukin-2 (IL-2) every week. PADRE-specific CD4+ T cells were generated from C57BL/6 mice immunized with pcDNA3-Ii-PADRE by gene gun. Cells were stimulated with irradiated PADRE peptide-pulsed DCs and IL-2 (10 IU) weekly [15].

### Peptide, antibodies and reagents

The PADRE peptide (Pan HLA-DR reactive epitope, AKFVAAWTLKAAA), and H2-D^b^-restricted HPV16 E7aa49–57 peptide (RAHYNIVTF) were synthesized by Beckman Coulter at a purity of 90%.

FITC, PE and APC-conjugated anti-mouse CD8a (clone 53.6.7), FITC-conjugated anti –mouse IFN-γ (clone XMG1.2), FITC and PE- conjugated anti-mouse CD4 (clone RM4-5), APC-conjugated anti-mouse CD11b (clone M1/70), PE-conjugated anti-mouse Ly6G (clone 1A8), PE-conjugated anti-mouse CD154 (CD40L, clone MR1), Functional anti-mouse CD95 (Fas, clone Jo2) agonistic antibodies, and FITC Annexin V Apoptosis Detection Kit, were purchased from BD Pharmingen (San Diego, CA). PE-conjugated CD178 (Fas-L, clone MFL3), PE-conjugated anti-mouse CD95 (Fas, clone 15A7), FITC-conjugated anti-mouse CD40 (cloneHM40-3), functional anti-mouse CD40 agonist (clone 1C10), and anti-mouse CD154 (CD40L, clone MR1) antibodies were purchased from eBioscience (San Diego, CA). PE-conjugated, HPV16 E7aa49–57 peptide and RAHYNIVTF loaded H2-D^b^ tetramers were obtained from National Institute of Allergy and Infectious Diseases tetramer Facility (Atlanta, GA). Fas/Fas L antagonist Kp7–6 was purchased from EMD Chemicals (San Diego, CA).

### *In vivo* tumor treatment experiments and antibody depletion

The tumor treatment experiments were performed twice independently. On day 0, 1×10^5^ TC-1 tumor cells were inoculated subcutaneously into C57BL/6 mice (5 per group). Four days later, tumor-bearing mice were treated with cisplatin (5 mg/kg body weight) or PBS control intraperitoneally. On day 5, mice were immunized intratumorally either with PBS control, 20 µg PADRE peptide, 10 µg CpG, or the combination of the latter two. All the treatments were repeated 3 more times at 7-day intervals. Tumor growth was monitored by visual inspection, palpation, and digital calipers (Scienceware) twice a week. Mice were sacrificed when the diameter of the tumor reached 20 mm.

For systemic antitumor assessment, mice (5 per group) were challenged subcutaneously with 1×10^5^ TC-1 tumor cells on the right flank. Five days later, 3×10^4^ TC-1 tumor cells were inoculated subcutaneously on the left flank. Tumor-bearing mice were either treated with PBS control or underwent triple therapy (cisplatin 5 mg/kg IP combined with 20 µg PADRE peptide and 10 µg CpG by intratumoral injection) three times at 5 days intervals. In the CD8+ T cell depletion group, 100 µg anti-mouse CD8 antibody (clone 2.43) was delivered to the mice via intraperitoneal injection on days 3, 4, 5 after tumor challenge. Subsequently, 150 µg/mouse anti-mouse CD8 antibody was delivered per week to maintain greater than 90% CD8+ T cell depletion.

### Preparation of single-cell suspensions from TC-1 subcutaneous tumor

The tumor tissues were gently dissected from the mice. The solid tumors were then minced into 1 to 2-mm pieces and incubated with serum-free RPMI 1640 medium containing 0.05 mg/ml collagenase I, 0.05 mg/ml collagenase IV, 0.025 mg/ml hyaluronidase IV, 0.25 mg/ml DNase I (both from Roche, Indianapolis, IN), 100 U/ml penicillin, and 100 µg/ml streptomycin and incubated at 37°C for 60 minutes as previously described [16].

### Cell surface staining, intracellular cytokine staining and flow cytometry analysis

For tetramer staining, single-cell suspended splenocytes or tumor infiltrating lymphocytes were stained with purified anti-mouse CD16/32 (Fc block, BD Pharmingen, San Diego, CA) first, and then with anti-mouse CD8-FITC, PE-conjugated HPV16 E7 aa49–57 (RAHYNIVTF) peptide-loaded H2-D^b^ tetramer at 4°C. The cells were stained with 7-AAD prior to flow cytometry analysis to exclude dead cells.

To detect HPV16 E7-specific CD8+ T cell responses and PADRE-specific CD4+ T cells by IFN-γ intracellular staining, PBMCs, single-cell suspended splenocytes or tumor infiltrating lymphocytes were stimulated with either HPV16 E7aa49–57 or PADRE peptide (1 µg/ml) in the presence of Golgiplug (BD Pharmingen, San Diego, CA) at 37°C overnight. The stimulated cells were then washed once with FACScan buffer and stained with PE-conjugated monoclonal rat anti-mouse CD8a or anti-mouse CD4. Cells were subjected to intracellular cytokine staining using the Cytofix/Cytoperm kit according to the manufacturer's instruction (BD Pharmingen, San Diego, CA). Intracellular IFN-γ was stained with FITC-conjugated rat anti-mouse IFN-γ. Flow cytometry analysis was performed using FACSalibur with CELLQuest software. All analyses were performed with Flowjo software (Tree star).

### Isolation of myeloid-derived suppressor cells in tumor-bearing mice and suppression of T cell proliferation

CD11b+ Ly6G^Hi^ MDSCs were isolated from single cell splenocyte suspensions of TC-1 tumor-bearing C57BL/6 mice using CD11b+Ly6G isolation kits and LS columns for magnetic separation according to the manufacture's instructions (Miltenyi Biotec). The purity was no less than 95%.

### *In vitro* MDSC apoptosis evaluation

Purified MDSCs were incubated at 37°C or co-cultured with PADRE-specific CD4+ T cells at a 1∶1 ratio for up to 24 hours in 96-well plates. Cells were then stained with APC anti-mouse CD11b, PE anti-mouse Ly6G, FITC anti-mouse Annexin V antibodies and 7-AAD (FITC Annexin V Apoptosis Detection Kit, BD Pharmingen, San Diego, CA) and examined by flow cytometry. MDSCs were first gated on CD11b+ and Ly6G^Hi^, and then the frequency of apoptosis was measured by Annexin V+ and 7-AAD.

To evaluate antibody induced MDSC apoptosis, isolated MDSC were cultured for 12 hours with anti-Fas agonistic antibody (2 µg/ml, BD Pharmingen, clone Jo2, San Diego, CA), or isotype mAb. For in vitro blocking assay, isolated MDSCs co-cultured with PADRE- specific CD4+T cell at 1∶1 ration, then incubated with isotype mAb, or Fas-Fas L antagonist (Kp7–6, CALBIOCHEM) for 24 hours.

### Statistical analysis

All data presented in this study are expressed as mean ± S.E. where indicated. Comparisons between individual data points for intracellular cytokine staining with flow cytometry analysis and tumor treatment were made using two-tailed student's t-test by Graph Prism 6.0. In the tumor treatment experiments, the principal outcome of interest was duration until mice were sacrificed. The event time distributions for different mice were compared using the Kaplan–Meier method and the log-rank test by Graph Prism 6.0. All p-values less than 0.05 were considered significant.

## Results

### Treatment of TC-1 tumor-bearing mice with cisplatin, CpG and PADRE peptide generates potent antitumor effects

**Figure 1:**
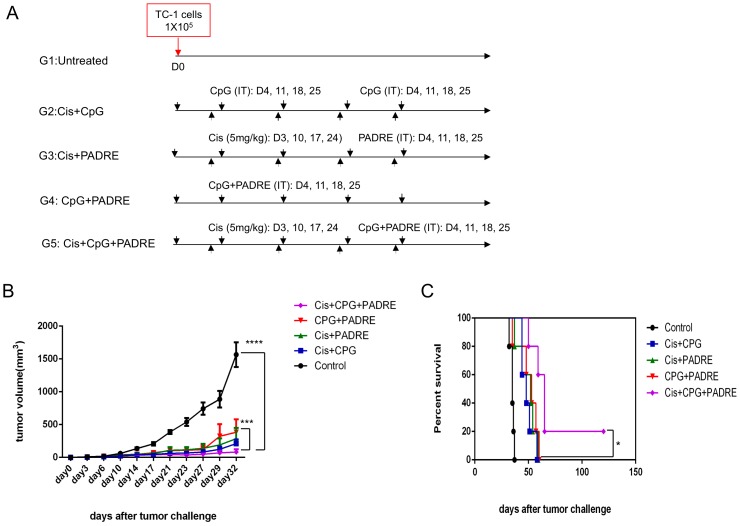
Antitumor effect in TC-1 tumor-bearing mice treated with intratumoral injection of irrelevant peptide combined with chemotherapy and CpG. C57BL/6 mice (5 mice/group) were challenged subcutaneously with TC-1 tumor cells and treated with various combinations of cisplatin, CpG and PADRE peptide. A. Schematic diagram of the treatment regimens. B. Plot of tumor growth kinetics. C. Kaplan-Meier survival plot. (*p<0.05, ***p<0.001, ***p<0.0001).

TC-1 tumor-bearing mice were used to examine the antitumor effects of different treatments combining intraperitoneal cisplatin chemotherapy, intratumoral injection of PADRE peptide and/or intratumoral injection of CpG. C57BL/6 mice were challenged with 1×10^5^ TC-1 tumor cells on day 0, and divided into five treatment groups. Mice in group 1 were untreated, those in group 2 were treated with cisplatin followed by intratumoral CpG, those in group 3 were treated with cisplatin followed by intratumoral PADRE peptide, those in group 4 were treated with intratumoral CpG and PADRE, and those in group 5 were treated with cisplatin followed by intratumoral CpG and PADRE (**Fig. 1A**). As shown in **Fig. 1B**, treatment with cisplatin followed by intratumoral CpG and PADRE significantly reduced tumor volume compared to control and treatment with CpG and PADRE only. Furthermore, treatment with cisplatin followed by intratumoral CpG and PADRE significantly improved survival compared to all other treatment groups (**Fig. 1C**). These data suggest that combination of cisplatin, CpG and PADRE has significant antitumor effects against TC-1 tumors.

### Treatment with cisplatin, CpG and PADRE peptide elicits strong systemic antitumor effects

**Figure 2:**
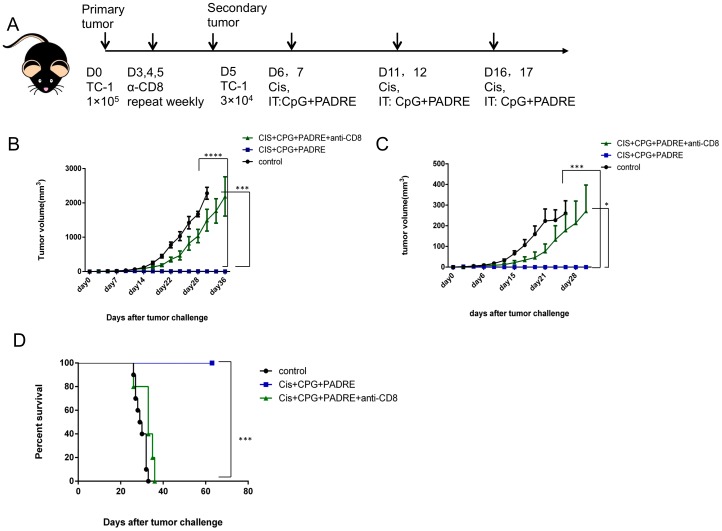
Local intratumoral treatment triggers systemic antitumor effects. C57BL/6 mice (5 mice/group) were challenged sequentially with TC-1 tumor cells on day 0 (right flank) and day 5 (left flank), and then were either untreated or treated with cisplatin combined with intratumoral injection with CpG and PADRE peptide in the primary tumor. In the CD8+ T cell depletion group, mice were treated as above, with the adition of 100 µg anti-mouse CD8 antibody (clone 2.43) injected intraperitoneally on days 3, 4 and 5 after tumor challenge, and then 150 µg per week subsequently. A. Schematic diagram of the treatment regimen. B. Scatter plot of primary tumor growth kinetics. C. Scatter plot of secondary tumor growth kinetics. D. Kaplan-Meier survival plot. (***p<0.001, ****p<0.0001).

Since treatment with cisplatin, CpG and PADRE generated the most potent antitumor effects (**Fig. 1** **)**, we next examined whether therapy with all three agents could generate antitumor effects in a secondary tumor. C57BL/6 mice were subcutaneously challenged with TC-1 tumor cells in the right flank on day 0, and then again on the left flank on day 5. A group of mice was then treated with anti-CD8 antibody on days 3-5 and then weekly to deplete CD8+ T cells. Mice were treated as shown in **Fig. 2A**, with cisplatin injected intraperitoneally and with CpG and PADRE injected intratumorally in the primary tumor on the right flank. As shown in **Fig. 2B**, the volume of the primary tumor was significantly decreased in mice treated with cisplatin, CpG and PADRE compared to untreated mice. Moreover, the volume of the secondary tumor on the left flank was also significantly decreased in mice treated with all three agents compared to untreated mice (**Fig. 2C**). Depletion of CD8+ T cells significantly decreased the therapeutic effect of cisplatin, CpG and PADRE treatment in both the primary and secondary tumors, indicating that CD8+ T cells are important for treatment efficacy (**Fig. 2B and C**). **Fig. 2D** shows that treatment with cisplatin, CpG and PADRE significantly prolongs the survival of TC-1 tumor-bearing mice compared to CD8+ T cell depleted mice and untreated mice. Similarly, depletion of CD4+ T cells significantly impaired the antitumor effects of the triple treatment regimen (**S1A Fig.**). Furthermore, infusion of PADRE-specific CD4+ T cells into tumor bearing mice significantly enhanced antitumor effect (**S1B Fig.**). These results show that CD4+ T cells are also important for the observed therapeutic effect. Taken together, the data indicate that treatment with cisplatin followed by CpG and PADRE generates potent systemic antitumor immune responses capable of acting against tumors at distant sites.

### Treatment with cisplatin, CpG and PADRE enhances systemic PADRE-specific CD4+ T cell responses in TC-1 tumor-bearing mice

**Figure 3:**
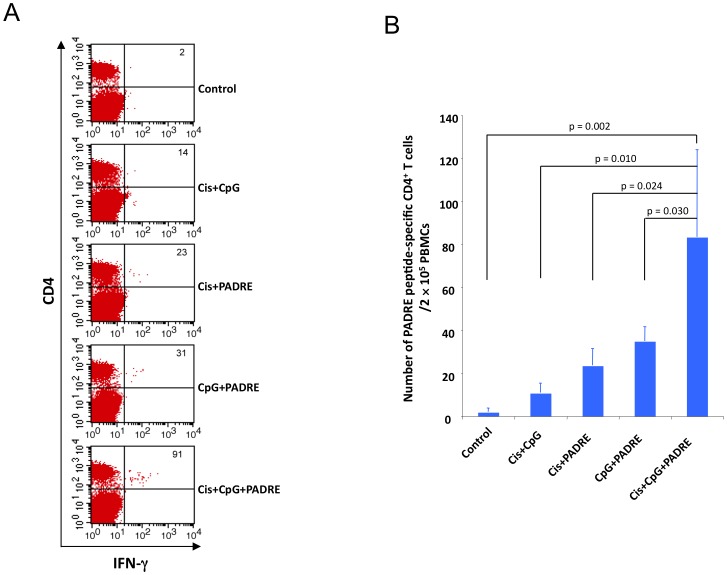
Systemic immune response to PADRE peptide. C57BL/6 mice (5 mice/ group) were challenged subcutaneously with TC-1 tumor cells and subsequently treated with various combinations of cisplatin, CpG and PADRE peptide as indicated. PBMCs were analyzed 1 week after the last antigen delivery. The presence of PADRE-specific CD4+ T cells in PBMCs was analyzed using intracellular cytokine staining for IFN-γ and CD4+ staining followed by flow cytometry analysis. A. Representative flow cytometry contour plot depicting the frequency of IFN-γ-secreting CD4+ T cells in the circulation after being pulsed with PADRE peptide. B. Bar graph quantification of the data (mean ±S.E.).

Next, we examined the effect of treatment with various combinations of cisplatin, CpG and PADRE on systemic CD4+ T cell immune responses. TC-1 tumor-bearing mice were treated with various combinations of cisplatin, CpG and PADRE peptide as shown in **Fig. 1A** and PBMCs were analyzed by flow cytometry 1 week after the last antigen delivery for the presence of IFN-γ secreting PADRE-specific CD4+ T cells. As shown in **Fig. 3A and B**, tumor-bearing mice treated with cisplatin followed by CpG and PADRE generated the highest number of PADRE-specific IFN-γ+ CD4+ T cells systemically compared to all other treatment groups. This indicates that treatment with cisplatin, CpG and PADRE is capable of generating potent PADRE-specific CD4+ T cell responses in TC-1 tumor-bearing mice.

### Treatment with cisplatin, CpG and PADRE induces local tumor antigen-specific CD8+ T cell responses in TC-1 tumor-bearing mice

**Figure 4:**
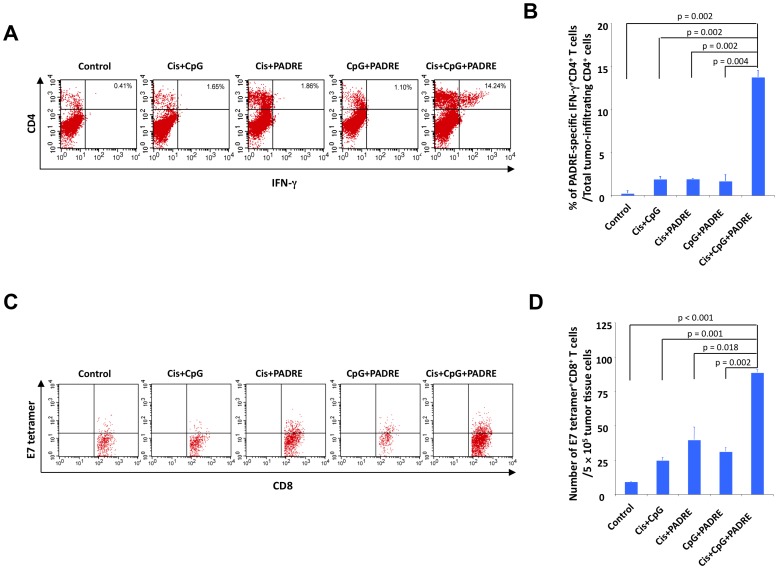
Local immune response to PADRE peptide and tumor antigen (HPV16 E7). C57BL/6 mice (5 per group) were challenged subcutaneously with TC-1 tumor cells and treated with various combinations of cisplatin, CpG and PADRE peptide as indicated. 12 days after the last antigen delivery, tumor-infiltrating lymphocytes were harvested to analyze the immune cell subsets. A. Representative flow cytometry analysis depicting the frequency of PADRE-specific IFN-γ-secreting CD4+ T cells among the total tumor infiltrating CD4+ T cells. B. Bar graph quantification of data from A. (mean ± S.E.). C. Representative flow cytometry analysis depicting the absolute number of E7 tetramer-binding CD8+ T cells in 5×10^5^ single-cell suspension prepared from tumors. D. Bar graph quantification of data from C. (mean ± S.E.).

TC-1 tumor-bearing mice were treated with various combinations of cisplatin, CpG and PADRE peptide as shown in **Fig. 1A**. Tumor-infiltrating lymphocytes (TIL) were harvested for analysis 12 days after the last antigen delivery. The presence of PADRE-specific CD4+ T cells and E7-specific CD8+ T cells among TILs was characterized using intracellular cytokine staining for IFN-γ as well as CD4 and CD8 staining and analyzed by flow cytometry. As shown in **Fig. 4A and C**, mice treated with cisplatin followed by CpG and PADRE generated the highest percentage of PADRE-specific CD4+ T cells among tumor-infiltrating CD4+ T cells compared to all other treatment groups. Furthermore, mice treated with cisplatin, CpG and PADRE generated the highest number of E7-specific CD8+ T cells among tumor cells compared to all other treatment groups (**Fig. 4B and D**). It is important to note that TILs collected from within the tumor microenvironment are in an activated state where the T cell receptors are being internalized. Thus, the staining appears as a gradient based on the various levels of TCR internalization. This appearance differs from the staining pattern usually observed in assessment of antigen-specific T cells in the systemic circulation, where a distinct population would be observed. Taken together, these data suggest that treatment with cisplatin, CpG and PADRE can lead to the cross-presentation of the tumor antigen, E7, resulting in enhanced tumor antigen-specific CD8+ T cell-mediated immune responses in the tumor loci.

### Treatment with cisplatin, CpG and PADRE decreases myeloid derived suppressor cells in tumor loci

**Figure 5:**
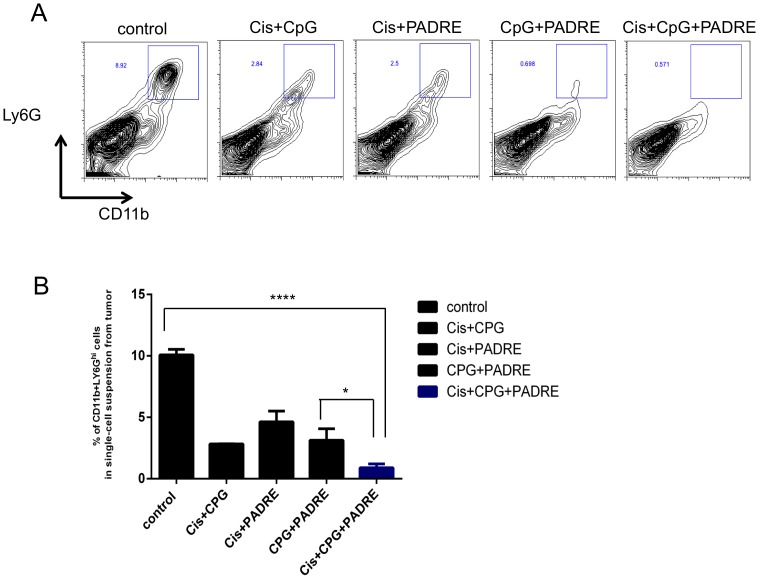
Intratumoral PADRE peptide injection combined with chemotherapy and CpG decreased the myeloid-derived suppressor cells in tumor loci. C57BL/6 mice (5 per group) were challenged subcutaneously with TC-1 tumor cells and treated with various combinations of cisplatin, CpG and PADRE peptide. At 30–35 days after tumor challenge, when the tumor diameters exceeded 10 mm, tumor-infiltrating cells were harvested to analyze for the presence of CD11b+ Ly6G^hi^ MDSCs. A. Representative contour plots depicting the frequency of CD11b+ Ly6G^hi^ MDSCs in single-cell suspension prepared from tumor. B. Bar graph quantification of data from A (mean ± S.E.) (*p<0.05, ***p<0.0001).

To further examine the effects of cisplatin, CpG and PADRE treatment, we analyzed the immunosuppressive myeloid derived suppressor cells (MDSCs) among tumor infiltrating-lymphocytes of treated TC-1 tumor-bearing mice. Between 30 and 35 days after tumor challenge, tumors were harvested and analyzed by flow cytometry as shown in **Fig. 5A**. **Fig. 5B** shows that tumors of mice treated with cisplatin, CpG and PADRE had a significantly lower percentage of CD11b+ Ly6G^Hi^ MDSCs in the tumor loci compared to all other treatment groups. These data suggest that treatment with cisplatin, CpG and PADRE elicits antitumor effects at least in part by reducing the population of MDSCs in the tumor microenvironment.

### PADRE-specific CD4+ T cells induces MDSC apoptosis through Fas and Fas ligand pathway

**Figure 6:**
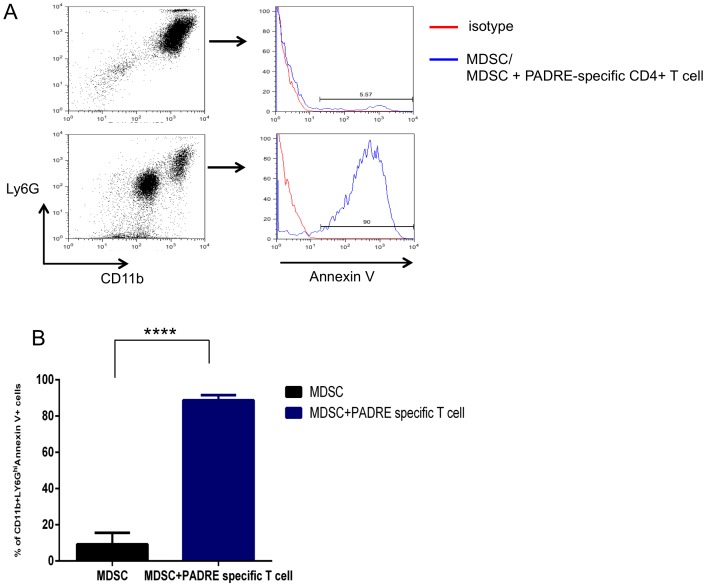
Activated CD4+ T cells directly induced apoptosis of MDSCs. Purified splenic CD11b+ Ly6G^Hi^ MDSCs were incubated with PADRE-specific CD4+ T cells or medium control for 24 hours. A. Representative flow cytometry plots depicting the frequency of CD11b+ Ly6G^Hi^ Annexin V+ MDSCs. B. Bar graph quantification of the data A (mean ± S.E.) (****p<0.0001).

**Figure 7:**
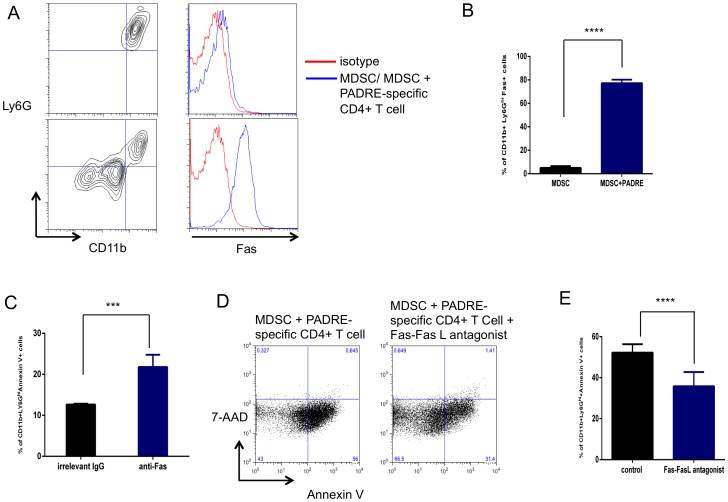
MDSCs underwent apoptosis via Fas/FasL-dependent pathway. A. Expression of Fas in MDSCs in the presence and absence of PADRE-specific CD4+ T cells. B. Bar graph quantification of the data in A (mean ± S.E.). C. Purified splenic Ly6G^Hi^ MDSCs were incubated for 12 hours with Fas agonist antibody (Jo2) or irrelevant control IgG, and the CD11b+ Ly6G^Hi^ cells were analyzed for the frequency of apoptosis (Annexin V+ and 7-AAD-). D. MDSCs were co-cultured with PADRE-specific CD4+ T cells for 24 hours combined with irrelevant control IgG or Fas-FasL antagonist Kp7–6. The CD11b+ Ly6G^Hi^ cells were analyzed for the frequency of apoptosis, characterized by Annexin V+ and 7-AAD-. Representative flow cytometry dot plots depicting the frequency of CD11b+ Ly6G^Hi^ Annexin V+ MDSCs. E. Bar graph quantification of the data in D (mean ± S.E.) (***p<0.001, ***p<0.0001).

We observed that treatment with cisplatin followed by CpG and PADRE reduced the number of MDSCs in tumor loci. Previously, it has been shown that MDSCs express Fas and will undergo Fas-FasL-mediated apoptosis if it encounters a T cell expressing FasL [17]. Therefore, we characterized the effect of activated PADRE-specific CD4+ T cells on MDSCs in terms of apoptotic activity and found that MDSCs incubated with PADRE-specific CD4+ T cells had significantly higher expression of annexin V compared to those incubated with medium control, indicating that activated CD4+ T cells are able to induce apoptosis of MDSCs (**Fig. 6**). Additionally, we observed that MDSCs co-cultured with PADRE-specific CD4+ T cells combined with Fas-FasL antagonist Kp7-6 significantly decreased the frequency of apoptosis compared to control (**Fig. 7**). Taken together, these data suggest that PADRE-specific CD4+ T cells induce apoptosis in MDSCs through a Fas-FasL-dependent pathway, indicating that the MDSC killing may occur via a non-antigen-specific pathway.

## Discussion

In the present study, we have shown that treatment with cisplatin followed by intratumoral injection of a potent adjuvant, CpG, and a Pan HLA-DR reactive epitope, PADRE peptide, generates potent antigen-specific cell-mediated immune responses and antitumor effects. Of note, treatment with cisplatin, CpG and PADRE generated systemic antitumor effects and was able to control tumors at distant sites. Furthermore, our treatment reduced the population of MDSCs in tumor loci, thereby reducing immunosuppression in the tumor microenvironment. Taken together, these data suggest that cisplatin, CpG and PADRE enhance the cross-presentation of tumor antigen to generate tumor antigen-specific CD8+ T cell-mediated immune responses. The combination of chemotherapy, adjuvant and immunogenic peptide presented here represents a universal approach to tumor control.

Of note, the current triple combination treatment approach does not include introduction of the E7 antigen through vaccination. Nevertheless, generation of E7-specific CD8+ T cells is observed (Fig. 4B). We reason that the observed E7-specific CD8+ T cell response is elicited by cross-presentation of the E7 antigens released from tumor following cisplatin-induced apoptosis by antigen presenting cells. Type 1 Interferon production following CpG treatment also serves to promote cross-presentation.

Here we observe that PADRE-specific CD4+ T cells are able to kill MDSCs through Fas-FasL interaction. In addition to PADRE-specific CD4+ T cells being able to kill MDSCs in the tumor microenvironment, any other activated T cells could also theoretically kill MDSCs. Indeed, we have recently shown that E7-specific CD8+ T cells and OT-1 T cells are capable of inducing apoptosis in MDSCs following treatment with cisplatin, CpG and GP33 peptide (Wu et al, personal communication). Once activated, CD4+ and CD8+ T cells release relevant cytokines such as IL-2, leading to upregulation of Fas expression on MDSCs. FasL is expressed by the activated T cells themselves. As a result, when an activated T cell encounters an MDSC, the MDSC will undergo Fas-FasL-mediated apoptosis [17]. The removal of the immunosuppressive MDSCs from the tumor microenvironment contributes to tumor control.

The current study serves as a platform that may be applicable to many patients with various cancers. A unique feature of the PADRE peptide is that the epitope it consists of is specific for many MHC class II molecules, and is therefore applicable to numerous individuals. Furthermore, we presume that this approach does not require identification of tumor antigen. The first round of treatment may induce tumor cell killing and the release of tumor antigen into the tumor microenvironment as well as the generation of PADRE-specific CD4+ T cells (**Fig. 3**). Following the subsequent rounds of treatment, the release of tumor antigen leads to the cross-priming and the generation of tumor antigen-specific CD8+ T cells, which are likewise able to kill tumor cells (**Fig. 4**). Due to this mechanism, it is not necessary to know immunodominant tumor antigens, making the approach potentially widely applicable.

Since the current approach involves intratumoral injection of therapeutic agents, it is most applicable to accessible tumors, such as head and neck, skin and cervical tumors. However, the approach may be limited in the case of inaccessible tumors, an issue that needs to be addressed in order to further extend this concept for clinical translation. This may be resolved by modifying the PADRE peptide to include tumor homing or tumor environment targeting aptamers. For example, CD13 ligand has been shown to be capable of delivering the antigenic peptide to tumor loci [18], [19] and elicit an antigen-specific antitumor response [1]. Additionally, we have shown that mesothelin and NKG2D can be used as tumor-homing modules in chimeric proteins, delivering the therapeutic proteins to tumor loci [20], [21]. Future studies are warranted to broaden the applicability of the current approach to inaccessible tumors.

The approach presented here may be an ideal candidate for clinical translation. Indeed PADRE and CpG have been tested in various clinical trials and found to be safe. PADRE has been tested in a formulation of a preventive cytomegalovirus (CMV) vaccine (NCT00722839) [22]. Additionally, PADRE is being tested in an ongoing clinical trial for breast cancer treatment as a part of the Mimotope P10s-PADRE/MONTANIDE ISA 51 VG vaccine (NCT01390064). CpG has been employed as a vaccine adjuvant in over 100 clinical trials (for review see [23]). Generally, vaccines containing CpG have been well tolerated. Furthermore, high doses (about 0.5 mg/kg) of free CpG used in cancer treatment have also been well tolerated in cancer patients [24]. It is also important to note that CpG and TLR9 interaction varies between different species due to the slight differences of TLR9 protein structures. Here we tested our combination approach with a CpG-motif and length most optimal for binding with mouse TLR9, which may not be the most optimal for human TLR9 interaction [25], [26]. For the combination approach to be translatable, CpG adjuvant most optimal for binding to human TLR9 will need to evaluated, and should be done in future studies.

In summary, we have successfully developed a strategy to control tumors using cisplatin treatment followed by intratumoral injection of CpG and PADRE peptide. This strategy may serve as a platform technology for the treatment of a variety of cancers, potentially including metastatic cancer. Additional studies will be needed to further enhance the stimulated immune responses and to extend the approach to inaccessible tumors.

## Supporting Information

S1 Fig **PADRE-specific CD4+ T cells contribute to antitumor effect.** A. C57BL/6 mice (5 per group) were challenged subcutaneously with TC-1 cells (10^5^ cells per mouse). 6 days after tumor challenge, cisplatin (5ug/gram of body weight) was injected intraperitoneally, followed by intratumoral delivery of PADRE peptide (20ug) and CpG (10ug) one day after. The treatment regimen was performed 3 times with a 5 day interval. For CD4 depletion, mice were injected with anti-CD4 antibody (100 ug) intraperitoneally daily for 3 days before the initial treatment followed by weekly injection. Graph showing the tumor growth kinetics. B. C57BL/6 mice (5 per group) were challenged subcutaneously with TC-1 cells (10^5^ cells per mouse). 4 days after tumor challenge, mice were treated with PADRE peptide (10 ug) twice a week. One group of mice also received intraperitoneal injection with PADRE-specific CD4+ T cells (2×10^6^) twice a week. Graph showing the tumor growth kinetics.
(TIF)
