# Supplementary figures and images for: Cancer Immunotherapy Employing an Innovative Strategy to Enhance CD4+ T Cell Help in the Tumor Microenvironment
Source: PLoS One. 2014 Dec 22;9(12):e115711. doi: 10.1371/journal.pone.0115711 (PMC4274108; doi:10.1371/journal.pone.0115711)

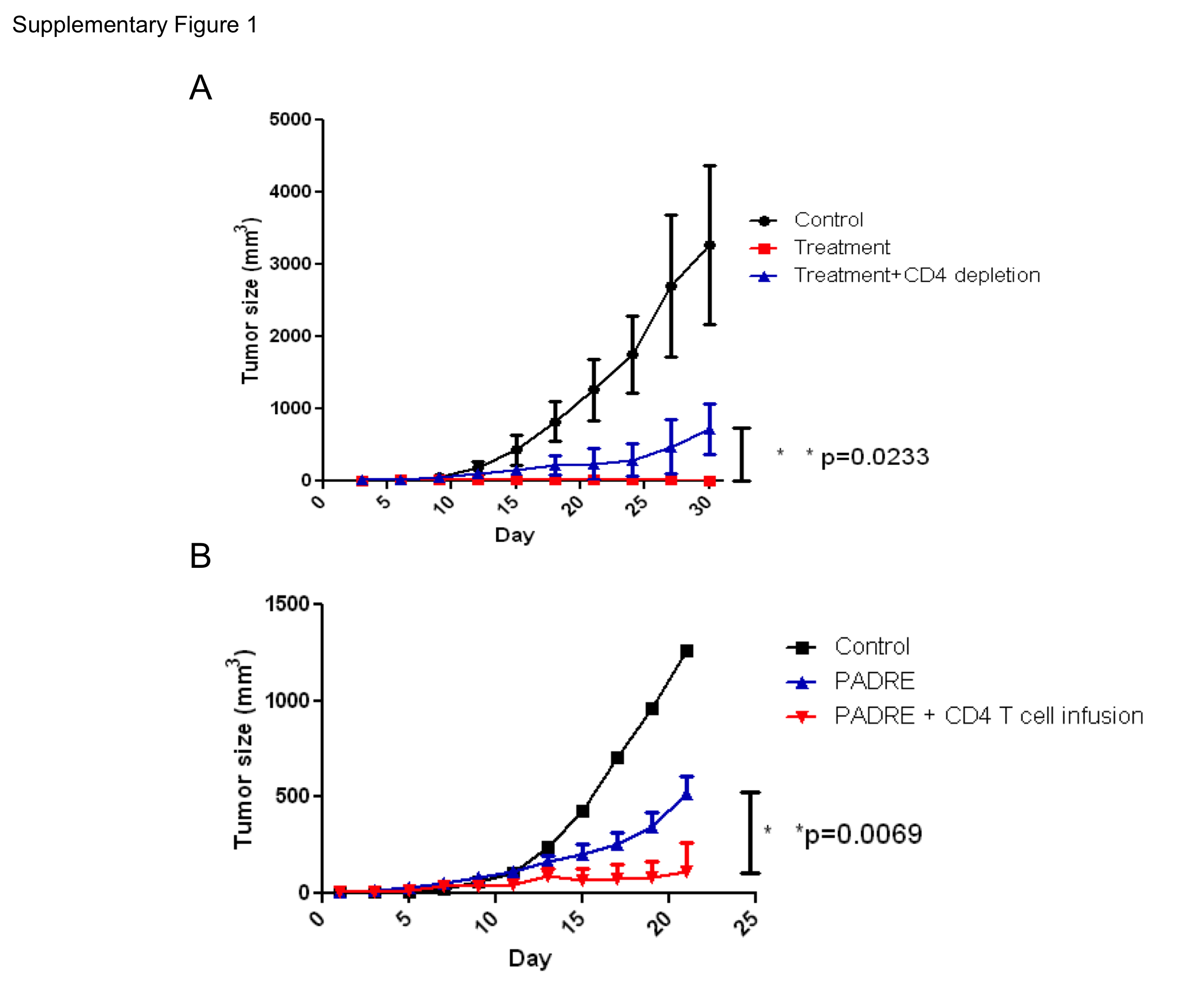

Supplement: S1 Fig — PADRE-specific CD4+ T cells contribute to antitumor effect. A. C57BL/6 mice (5 per group) were challenged subcutaneously with TC-1 cells (105 cells per mouse). 6 days after tumor challenge, cisplatin (5ug/gram of body weight) was injected intraperitoneally, followed by intratumoral delivery of PADRE peptide (20ug) and CpG (10ug) one day after. The treatment regimen was performed 3 times with a 5 day interval. For CD4 depletion, mice were injected with anti-CD4 antibody (100 ug) intraperitoneally daily for 3 days before the initial treatment followed by weekly injection. Graph showing the tumor growth kinetics. B. C57BL/6 mice (5 per group) were challenged subcutaneously with TC-1 cells (105 cells per mouse). 4 days after tumor challenge, mice were treated with PADRE peptide (10 ug) twice a week. One group of mice also received intraperitoneal injection with PADRE-specific CD4+ T cells (2×10^6^) twice a week. Graph showing the tumor growth kinetics. (TIF) [file pone.0115711.s001.tif]
